# Symptoms of posttraumatic stress disorder in a clinical sample of refugees: a network analysis

**DOI:** 10.1080/20008198.2017.1318032

**Published:** 2017-05-16

**Authors:** Tobias R. Spiller, Matthis Schick, Ulrich Schnyder, Richard A. Bryant, Angela Nickerson, Naser Morina

**Affiliations:** ^a^ Department of Psychiatry and Psychotherapy, University Hospital, University of Zurich, Zurich, Switzerland; ^b^ School of Psychology, UNSW Australia, Sydney, Australia

**Keywords:** Network analysis, symptomics, PTSD, refugees

## Abstract

**Background**: Network analysis is an emerging methodology for investigating psychopathological symptoms. Given the unprecedented number of refugees and the increased prevalence of mental disorders such as posttraumatic stress disorder (PTSD) in this population, new methodologies that help us better to understand psychopathology in refugees are crucial.

**Objective**: The objective of this study was to explore the network structure and centrality indices of DSM-5 PTSD symptoms in a cross-sectional clinical sample of 151 severely traumatized refugees with and without a formal PTSD diagnosis.

**Method**: The R-packages *qgraph* and *bootnet* were used to estimate the structure of a PTSD symptom network and its centrality indices. In addition, robustness and significance analyses for the edges weights and the order of centrality were performed.

**Results**: Three pairs of symptoms showed significantly stronger connections than at least half of the other connections: hypervigilance and exaggerated startle response, intrusion and difficulties falling asleep, and irritability or outbursts of anger and self-destructive or reckless behaviour. Emotional cue reactivity had the highest centrality and trauma-related amnesia the lowest.

**Conclusion**: Although only 51.0% of participants fulfilled criteria for a probable PTSD diagnosis, emotional cue reactivity showed the highest centrality, emphasizing the importance of emotional trauma reminders in severely traumatized refugees attending an outpatient clinic. However, due to the small sample size, the results should be interpreted with care.

## 1. Introduction

Network analysis is a graph theory based methodology which can be used to analyse the relationship between observable variables, including symptoms of psychopathology (Borsboom & Cramer, 2013). The underlying rationale is that symptoms of mental health disorders are causally mutually dependent and thus influence each other (Cramer, Waldorp, van der Maas, & Borsboom, 2010; McNally, 2016). This is in contrast to the model of psychopathology used by current nosological systems such as DSM-5 (American Psychiatric Association, 2013) or ICD 10 (World Health Organization, 1992), in which symptoms are considered to be causally independent of each other and as being the result of an unobserved, latent entity (Hofmann, Curtiss, & McNally, 2016). This model has clinical and scientific importance, shifting the focus from finding the underlying cause of a defined psychopathological syndrome to the investigation of the symptoms’ interdependent relationships (Fried, 2015).

To the best of our knowledge, only three studies conducting network analysis with posttraumatic stress disorder (PTSD) symptoms have been published to date. In the first study, McNally et al. (2015) performed a DSM-IV PTSD symptom network analysis in a sample of earthquake survivors in China (McNally et al., 2015). They reported strong connections between several symptoms: avoidance of thoughts and avoidance of activities; traumatic dreams, intrusions and flashbacks; and exaggerated startle response and hypervigilance. The most central symptom in the network was traumatic dreams, the least central trauma-related amnesia. A second study analysed the network structure and centrality of DSM-5 PTSD symptoms and incorporated variables (e.g. suicidal ideation) in a sample of U.S. veterans (Armour, Fried, Deserno, Tsai, & Pietrzak, 2017). Key findings included, among others, strong connections between flashbacks and nightmares; hypervigilance and exaggerated startle response; and detachment and restricted affect, high centrality measures for negative trauma-related emotions and physiological cue reactivity and a low centrality for trauma-related amnesia. A third study analysed PTSD symptoms in traumatic injury survivors in the week following trauma, and again 12 months later (Bryant et al., 2017). This study found that re-experiencing symptoms formed a distinct network in the acute phase, and that network connectivity was significantly stronger in the chronic relative to the acute phase.

To date, no studies have applied network analysis approaches to a refugee population. Currently, the number of refugees is the highest since WWII (UNHCR, 2016). Refugees are commonly exposed to a large number of potentially traumatic events, which is associated with an increased risk for mental health disorders such as PTSD and depression (Mollica et al., 1998; Silove et al., 2014). In addition, in refugees it is common that several years have passed since the onset of PTSD symptoms and the initiation of therapy (Schick et al., 2016), with PTSD persisting during this period. Network analysis, with its focus on the relationship between symptoms, may help to improve the understanding of symptom interactions and, when investigating longitudinal data, their contribution to the maintenance of PTSD.

The purpose of the current study was to explore the network structure and centrality of DSM-5 PTSD symptoms in a cross-sectional clinical sample of refugees attending two outpatient clinics for victims of torture and war in Switzerland. Based on the existing studies that have used network analysis, we generated tentative hypotheses about potential results. We first hypothesized strong connections between hypervigilance and exaggerated startle response, and between flashbacks and nightmares. Second, as all participants in this study were treatment-seeking at one of two outpatient clinics for victims of war and torture, we expected high centrality measures for symptoms grouped in the re-experiencing cluster of DSM-5 PTSD diagnosis (i.e. intrusions), reflecting a high load of pathognomonic symptoms of PTSD. Based on the study by Armour et al. (2017), we also predicted high centrality measures for symptoms grouped in the cluster describing negative alterations in cognitions and mood (i.e. strong general negative emotional state).

## 2. Methods

### 2.1. Participants

All participants were asylum seekers or refugees in treatment, recruited from two outpatient units for victims of torture and war, in Zurich and Bern, Switzerland. Inclusion criteria required speaking one of the study languages (German, Turkish, Farsi, Arabic, Tamil or English) and being at least 18 years old. Exclusion criteria were acute suicidality, severe dissociative symptoms and current psychotic symptoms based on the clinical judgment of the therapist in charge. This led to the invitation of a total of 172 patients, 154 of whom (89.5%) agreed to participate. Three of these patients failed to attend or complete the research session, resulting in the assessment of 151 participants.

### 2.2. Measures

Accredited interpreters translated and back-translated all measures used for this study following gold standard translation guidelines (Bontempo, 1993). Discrepancies were revised by independent bilingual individuals familiar with health-related questionnaires in cooperation with the research team.

#### 2.2.1. Trauma exposure

To index trauma exposure, a combined version of the Harvard Trauma Questionnaire (HTQ; Mollica et al., 1992) and the PDS trauma checklist (Foa, Cashman, Jaycox, & Perry, 1997) was used. In research with refugees, the HTQ was previously used to index trauma exposure (De Fouchier et al., 2012; Ekstrøm, Carlsson, Sonne, & Mortensen, 2016). Overall trauma exposure was calculated as the number of different types of traumatic events experienced and/or witnessed by each participant (ranging from 0 to 23).

#### 2.2.2. PTSD symptoms

Symptoms of PTSD were assessed using the third part of the Posttraumatic Diagnostic Scale (PDS; Foa et al., 1997). The PDS contains 17 items assessing the occurrence of DSM-IV symptoms of PTSD in the previous four weeks on a 4-point Likert-scale (0 = *not at all or only one time*, 3 = *5 or more times a week/almost always*), unanswered items were coded as missing. To account for the anticipated changes of the diagnostic criteria of PTSD in DSM-5 (American Psychiatric Association, 2013), which was not yet published at the beginning of the data collection, four additional items were added (Persistent negative beliefs about yourself, others, or the world; Persistent extreme blame of yourself or others for what happened; Strong general negative emotional state; Excessive risk-taking or doing things that might hurt you) and one item was excluded, as it was no longer included in the DSM-5 diagnostic criteria (Sense of a foreshortened future). A comparison of DSM-IV and DSM-5 diagnostic criteria using part of this sample has been published elsewhere (Schnyder et al., 2015). We computed a total continuous score of PTSD symptoms (Range 0–60) and employed a DSM-5 based diagnostic algorithm to determine likely PTSD caseness. Specifically, a symptom was considered to be present if the participants rated it as 2 or 3. Thus, consistent with DSM-5 criteria, a participant was considered to have a likely PTSD caseness if he or she reported one or more intrusion symptoms, one or more avoidance symptoms, two or more negative alterations in cognition and mood, and two or more alterations in arousal and reactivity. The scale in this study showed strong internal consistency with α = 0.91. Assessing PTSD symptoms and PTSD diagnosis using the PDS has been previously used in research with refugees (Neuner, Schauer, Klaschik, Karunakara, & Elbert, 2004; Turner, Bowie, Dunn, Shapo, & Yule, 2003).

### 2.3. Procedures

The study was approved by the Ethics Committees of the Cantons of Zurich and Bern, Switzerland. Prior to the assessment, a study team member explained the purpose of the study to each participant. Next, participants willing to participate were informed about the option of withdrawing from the study at any time without negative consequences, and written informed consent was obtained. The assessment of self-reported questionnaires was conducted with a computer-based assessment tool (MultiCasi; Knaevelsrud & Müller, 2008) with assistance from a psychiatrist, clinical psychologist or a master-level student of clinical psychology. Reimbursement for participants was CHF 40 (approximately US$40).

### 2.4. Data analysis

Participant characteristics were analysed using SPSS IBM Inc. Version 23 (IBM Corp., Released 2014). The *R*-package *qgraph* (Epskamp, Cramer, Waldorp, Schmittmann, & Borsboom, 2012) was used to estimate the structure of the network and the centrality of symptoms. To analyse robustness of our estimations, we used the *R*-package *bootnet* (Epskamp, Borsboom, & Fried, 2017). The visualization of the network was made using *qgraph* and checked with *bootnet*, which showed matching results. The analytic process followed the recommendations on psychological network analysis written by the developers of the *R*-packages, which also includes further information about the analytic methods used and the conceptual reasoning behind it (Epskamp et al., 2017).

#### 2.4.1. Missing data

A total of 1.8% of the data was missing. To handle missing data, we used the pairwise deletion built in to qgraph.

#### 2.4.2. Network estimation and visualization

The form of representation of symptoms and their associations as a network is most often a network of partial correlation coefficients, also called Gaussian Graphical Model. In the form of presentation that we chose for our study, each of the 20 nodes represents one of the 20 symptoms of the DSM-5 definition of PTSD. Edges represent a weighted connection between two symptoms after controlling for all other edges in the network and can be understood as partial correlation coefficients. These weights range from −1 to 1 and are also called conditional independence associations. In a first step, a correlation matrix was computed. As recommended for ordinal and skewed data structure, the matrix was based on polychromic correlations (Epskamp et al., 2017). Second, the network was estimated using EBICglasso, an estimation procedure to minimize false positive detection of connections adapted from the LASSO regularization method (Tibshirani, 1996). Third, in order to visualize the estimated network, the Fruchterman-Reingold algorithm (Fruchterman & Reingold, 1991), which places nodes with more and/or stronger connections more closely together, was used.

#### 2.4.3. Centrality estimation

Centrality estimation was conducted to identify the relevance of the single symptoms for the network structure. Three different centrality measures were estimated: strength, closeness and betweenness. Node strength estimates the direct connection of a node to a network by summing all weights of each edge of a node. Closeness estimates the connection of a node to a network indirectly based on the average distance from a node to all other nodes in a network by using the inverse of shortest path length. Betweenness is also an indirect measurement for centrality and is the number of times a node is on the shortest path between any two nodes. The higher the degree of a centrality measure, the more central a given node is in a network (more information about centrality measurements can be found here: Opsahl, Agneessens, & Skvoretz, 2010).

#### 2.4.4. Robustness estimation and testing for significance

Currently, the robustness (accuracy and stability) of estimating a symptom-based network structure is still a major challenge (Epskamp et al., 2017). As recommended at the time of the data analysis, we used bootstrap confidence regions to examine the certainty of the edges and tested for significance between edge-weights with α = 0.05 based on 1000 bootstrap iterations. To estimate the stability of the order of the centrality indices, we used a node-dropping sub-setting bootstrap technique and the CS-coefficient, which is an index for stability of the centrality indices (although there is no strict cut-off, its value should be at least 0.25, preferably higher than 0.5 ; Epskamp et al., 2017). Furthermore, we tested node strength for significance with α = 0.05 based on 1000 bootstrap iterations

## 3. Results

### 3.1. Sample characteristics

A total of 70% (*N* = 106) of the participants were male and the mean age was 41.9 years (*SD* = 9.8). Participants came from a variety of countries of origin, including Turkey (54%, *N* = 81), Iran (9%, *N* = 13), Sri Lanka (9%, *N* = 13), Afghanistan (7%, *N* = 7), Bosnia (4%, *N* = 6) and others (21%, *N* = 31). Participants had experienced a mean of 14.7 (*SD* = 4.1) different types of potentially traumatic events. Concerning the residency status, 39% (*N* = 58) had an insecure visa status, 60% (*N* = 91) had a secure visa status or were naturalized Swiss citizens, and in 1% (*N* = 2) the visa status was unknown. A total of 51.0% (*N* = 77) fulfilled criteria for a probable PTSD diagnosis, and the mean PDS score was 37.88 (*SD *= 8.01). The means and standard deviations of symptom endorsements are shown in Table 1.

**Table 1.** Single symptom endorsements, means and standard deviations.

ID	Mean	Standard deviation
FLASH	2.03	.92
DREAM	1.86	1.00
INTRU	1.71	1.00
EREAC	1.95	.99
PREAC	1.78	1.01
AVTHT	1.75	1.03
AVSIT	1.70	1.08
AMNES	1.24	1.10
DISINT	1.44	1.10
DTACH	1.74	1.11
RAFF	1.73	1.11
SLEEP	2.05	1.03
ANGE	1.53	1.06
CONC	2.13	.93
HYPER	1.75	1.08
STRTL	1.82	.99
NBEL	1.67	1.11
BLAM	1.67	1.04
NEMO	1.68	1.02
RISK	.99	1.03

ID	Variable
FLASH	Recurrent and intrusive distressing recollections of the event
DREAM	Recurrent distressing dreams of the event
INTRU	Acting or feeling as if the traumatic event were recurring
EREAC	Emotionally upset when reminded of the traumatic event (emotional cue reactivity)
PREAC	Experiencing physical reactions reminded of the traumatic event (physical cue reactivity)
AVTHT	Avoid thoughts, feelings or conversations associated with the trauma
AVSIT	Avoid activities, people or places reminding of the traumatic event
AMNES	Inability to recall an important aspect of the trauma (trauma-related amnesia)
DISINT	Markedly diminished interest or participation in significant activities
DTACH	Feeling of detachment or estrangement from others
RAFF	Restricted range of affect
SLEEP	Difficulty falling or staying asleep
ANGE	Irritability or outbursts of anger
CONC	Difficulty concentrating
HYPER	Hypervigilance
STRTL	Exaggerated startle response
NBEL	Persistent negative beliefs about oneself, others or the world
BLAM	Persistent extreme blame of oneself or others for what happened
NEMO	Strong general negative emotional state
RISK	Excessive risk-taking or doing things that might hurt oneself (self-destructive or reckless behaviour)

Brackets: Due to clarity and simplicity of the text, some items were used under the term set in brackets (i.e. ‘Inability to recall an important aspect of the trauma’ was used as ‘Trauma-related amnesia’).

### 3.2. Network and centrality analysis

#### 3.2.1. Network

**Figure 1.**
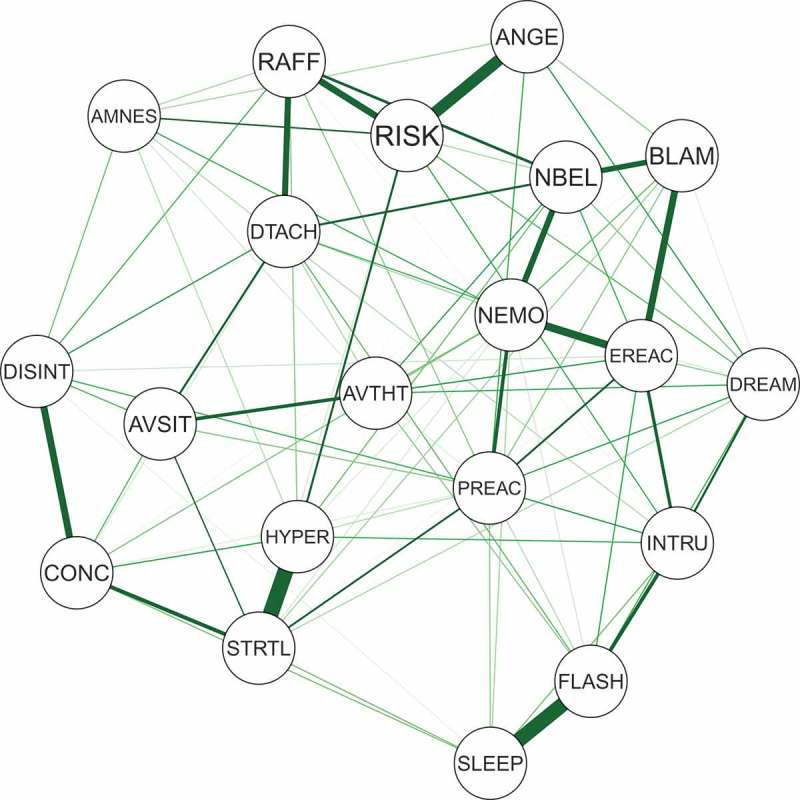
Estimated network of 20 DSM-5 PTSD symptoms. Nodes represent symptom and edges represent a partial correlation between the symptoms, after controlling for all other correlations of a given node. PTSD: Posttraumatic stress disorder; DSM-5: Diagnostic and Statistical Manual of Mental Disorders, Fifth Edition.

**Figure 2.**
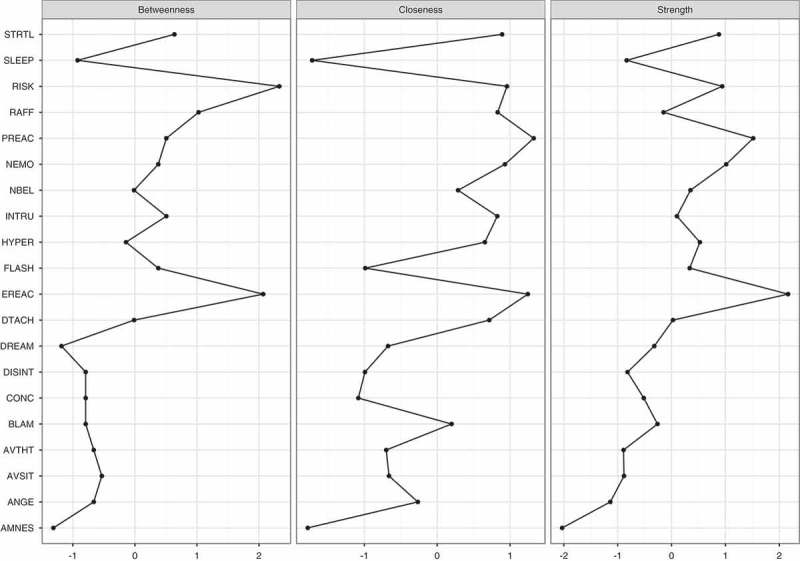
Centrality measurements for the estimated network of 20 DSM-5 PTSD shown in Figure 1. PTSD: Posttraumatic stress disorder; DSM-5: Diagnostic and Statistical Manual of Mental Disorders, Fifth Edition.

The network structure of the 20 DSM-5 PTSD symptoms is depicted in Figure 1. In the visual analysis of this network, only positive connections emerged, indicating a network with several dense connections. Bootstrap confidence regions for the edges weights were mostly overlapping (shown in Figure 2), and the bootstrap difference test revealed that only three connections differed significantly from at least half of the other edges, namely (a) between hypervigilance and exaggerated startle response, (b) intrusion and difficulties falling asleep, and (c) irritability or outbursts of anger and self-destructive or reckless behaviour (shown in Figure S1 in Supplemental data).

#### 3.2.2. Centrality measures

**Figure 3.**
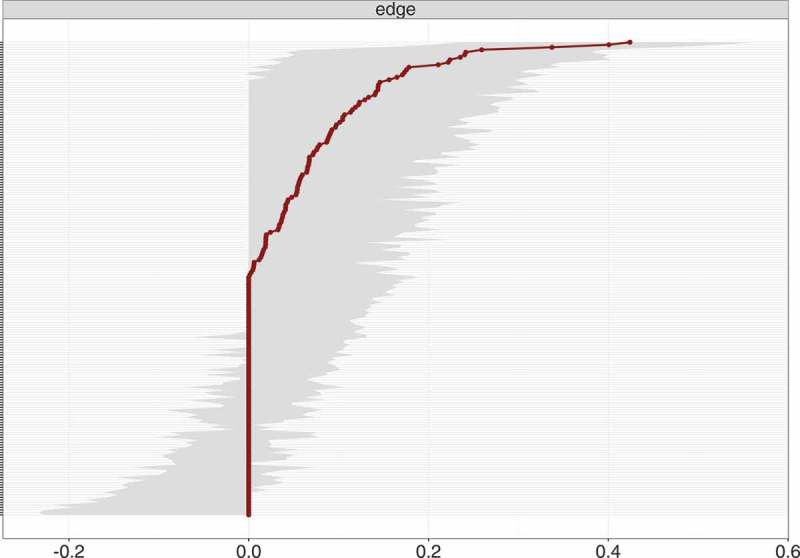
Bootstrap 95% confidence intervals for estimated edge weights for the network of 20 DSM-5 PTSD symptoms shown in Figure 1. The edge weights, each horizontal line representing one edge, are represented by the red line, the 95% confidence intervals by the grey area. PTSD: Posttraumatic stress disorder; DSM-5: Diagnostic and Statistical Manual of Mental Disorders, Fifth Edition.

**Figure 4.**
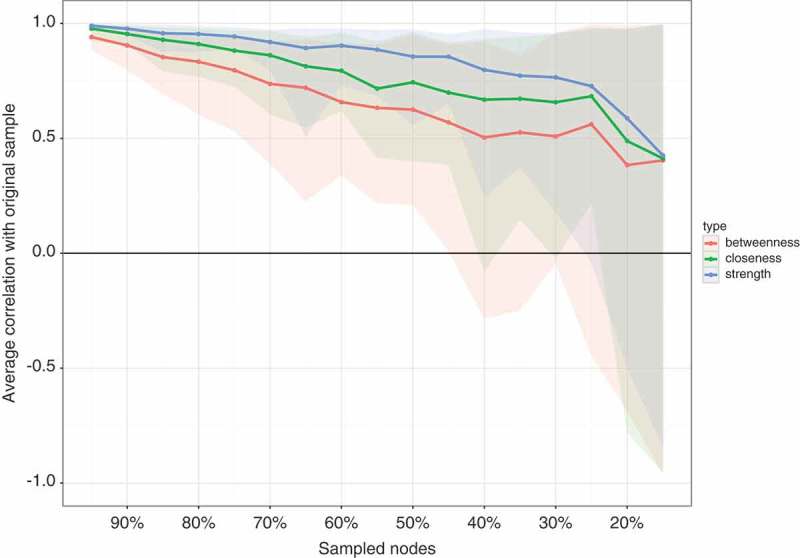
The average correlation between bootstrap centrality measures of networks sampled with node-dropping and the network of 20 DSM-5 PTSD symptoms shown in Figure 1. PTSD: Posttraumatic stress disorder; DSM-5: Diagnostic and Statistical Manual of Mental Disorders, Fifth Edition.

The standardized centrality indices are shown in Figure 3. The result of the node-dropping bootstrap technique to estimate the stability of the centrality indices is shown in Figure 4. Robustness analyses of the centrality indices showed a CS-coefficient of 0.45 for strength, 0.25 for closeness and 0.15 for betweenness. With node strength being the most reliable centrality index, the interpretation of each symptom’s relevance is based on node strength alone. The bootstrap difference test showed that only node strength for emotional cue reactivity differed significantly from other nodes (shown in Figure S2 in Supplemental data).

## 4. Discussion

This study is, to our knowledge, the first to present a network analysis of PTSD symptoms in refugees. We found partial support for our first hypothesis, namely a strong connection between hypervigilance and exaggerated startle response, but not between flashbacks and nightmares. Regarding our second hypothesis, robustness analysis showed instable results. However, partly consistent was the finding of emotional cue reactivity showing the highest centrality in visual inspection. Nonetheless, due to the relatively small sample size, the power and robustness of our analysis are low and therefore the results should be interpreted with care.

Consistent with our first hypothesis was the strong connection between hypervigilance and exaggerated startle response. Significance testing revealed that only this connection and the one between intrusion and difficulties falling asleep differed from most other connections (see Figure S1 in Supplemental data) and that only the connection between outbursts of anger and self-destructive or reckless behaviour differed from at least half of all other connections. Given this, the accuracy of the visual interpretation of strength of edges in our network analysis and the comparability of these results with previous findings by McNally et al. (2015), Armour et al. (2017) and Bryant et al. (2017) is limited (note that due to the characteristics of the network estimation’s procedure all edges shown are themselves significant, and only differences in weight was tested). However, a strong connection between hypervigilance and exaggerated startle response was reported in all three other studies, whereas the one between intrusion and difficulties falling asleep was not observed as being strong (Armour et al., 2017) or present (Bryant et al., 2017; McNally et al., 2015). This incongruent finding could be the result of different populations, exposure to different types of trauma (military personal and refugees versus civilian and natural disaster) or a false positive result due to the relatively small sample size in Armour et al. (2017) as well as in our study. Nevertheless, from a clinical perspective, both findings are not surprising. Although these associations seem straightforward, it is possible that they are more complex. For example, the association between intrusions and difficulties falling asleep could be influenced by, or even be the result of, rumination (Steil & Ehlers, 2000). Therefore, additional research is needed to investigate the associations of these symptoms in greater depth, especially in studies trying to unveil causal relations.

Visual inspection of centrality partially supported our second hypothesis. In contrast, centrality analysis revealed unstable results with the CS-coefficient for node strength being 0.45 and the bootstrapped difference test showing that only emotional cue reactivity had significantly higher strength compared to some, but not all, nodes. Thus, our results require careful interpretation. Furthermore, recent work has shown that network structure can be driven by differences in item variance (Terluin, De Boer, & de Vet, 2016). Regarding our results, emotional cue reactivity had a similar mean and standard deviation to other items, making it unlikely that its centrality was mainly the result of differences in item variance. However, in accordance with the methodology of previous studies, visual inspection of node strength showed the highest strength for emotional cue reactivity, followed by physical cue reactivity (both from cluster B), high centrality of self-destructive behaviour, a strong general negative emotional state and exaggerated startle response. The predominance of centrality of emotional and physical cue reactivity was impressive, especially given that only 51.0% of our participants fulfilled the criteria for a probable PTSD diagnosis. Nevertheless, finding re-experiencing symptoms to have high importance for symptom structure in treatment-seeking refugees was in accordance with our clinical experience and showed the high symptomatic burden of our patients and the need for treatment, which was also reflected by the high centrality of self-destructive behaviour. Given the association with physical cue reactivity, the finding exaggerated startle response having high centrality was not surprising. The high centrality of a strong general negative emotional state was also consistent with the findings of Armour et al. (2017) and our clinical experience and may be explained by the common co-occurrence of depression in refugees with PTSD (Momartin, Silove, Manicavasagar, & Steel, 2004). Due to the relatively small sample size, we did not include depression symptoms in our analysis. Investigating the co-morbidity of PTSD and depression in larger samples in future studies would be important. Furthermore, there may be moderators and mediators for the association between trauma exposure and network structure of PTSD and depression symptoms, influencing centrality indices. For example, Nickerson et al. (2015) reported that emotion regulation difficulties mediated the association between trauma exposure and psychological symptoms (Nickerson et al., 2015). Future research should investigate these interactions in detail.

There were also conflicting results with the three prior studies. McNally et al. (2015) and Bryant et al. (2017) reported that ‘feeling that your future will be cut short’ and ‘concentration difficulties’ showed high centrality. These differences are partly the result of different PTSD diagnostic criteria that were used (‘feeling that your future will be cut short’ is no longer included in the DSM-5 definition of PTSD, while the item ‘strong general negative emotional state’ was added to the definition; American Psychiatric Association, 2013). The different trauma-types experienced and the different settings in which participants were recruited (part of a clinical research sample versus treatment-seeking) may also have contributed to these differences. In addition, the lack of power and the low robustness of our findings, resulting from of our small sample size, may have contributed to this discrepancy as well. In contrast to Armour et al.’s (2017) study, we did not find high centrality for detachment. However, the reporting of three out of five (Armour et al., 2017; Bryant et al., 2017), respectively two out of five (McNally et al., 2015) symptoms of the re-experiencing cluster having high centrality, the high relevance of these symptoms for network structure of PTSD symptoms was a similar finding in these three studies and our own investigation.

It is noteworthy that reckless behaviour was a highly central symptom. Reckless behaviour and self-destructive tendencies are a major clinical concern in clinical populations, and there is an urgent need to better understand the factors that influence these reactions. This finding contrasted with the three other studies, which found reckless behaviour to have only moderate centrality (Armour et al., 2017; Bryant et al., 2017; McNally et al., 2015). Anger is especially prominent in victims of torture and veterans (Orth & Wieland, 2006), therefore this finding may partly result from the different samples used in the studies. Nevertheless, the stability of our results, as well as the low power of our study, could also have influenced our results; consequently, this finding should be interpreted with care.

We also note that trauma-related amnesia showed the lowest centrality on visual inspection. This result was in line with the other three studies conducted by McNally et al. (2015), Armour et al. (2017) and Bryant et al. (2017). Again, given the limited robustness of our findings and the low power of our study, our results should be replicated with a larger sample size.

### 4.1. Limitations and future directions

This study has several limitations. First, for the given number of variables the number of parameters estimated exceeded the sample size. This is an important limitation of our study resulting in low power and low robustness of our results. Therefore, the validity of the visual interpretation of edge weight and node strength is questionable. Consequently, the interpretation should be done most carefully. Hence, replicating this study with a larger sample is crucial. In addition, standards for conductance and interpretation of significance and robustness tests are currently not formally established. Developing these standards is of high importance and could render the interpretation of our results more effective. Second, the sample included participants with and without a formal PTSD diagnosis, which limits the generalizability of our findings. Nonetheless, the objective of this study was to investigate PTSD symptom network structure in a clinical environment, where severely traumatized patients were also treated when having a high burden of pathology without fulfilling formal PTSD criteria. Third, the cross-sectional nature of our data, the gender imbalance, and the variety of participants’ cultural backgrounds further limits generalization to other populations and settings. Importantly, the nature of network analysis does not permit causal inferences. However, the clinical nature of our sample is also a strength as it presents a close-to-reality scenario.

With regard to further avenues for research, we suggest that four major points require investigation. First, given the limited robustness of our results, replicating our study with a much larger sample is crucial to increase the reliability of findings. Second, a larger sample would also allow for the investigation of co-morbidity. As the co-occurrence of PTSD and depression is common in severely traumatized refugees (Silove et al., 2014), exploring the associations between PTSD and depression symptoms with network analysis may improve our understanding of the co-occurrence and interactions of these two disorders. Third, network structure and centrality measures in different sub-samples, i.e. based on gender or visa status, could be studied and compared with each other in larger samples as well. Fourth, only the investigation of longitudinal data will ultimately unveil the dynamics of PTSD symptom interaction. This is crucial if network analysis is used to help identifying symptom interactions reinforcing or weakening each other, or how treatment changes the PTSD symptom network. For this purpose, studying changes and stability in symptom networks over time, i.e. during a waitlist period, or before and after therapeutic intervention, would be of key interest.

### 4.2. Conclusion

The current study was the first to investigate PTSD symptom networks in a clinical sample of severely traumatized refugees. The results showed that two pairs of symptoms were connected more strongly than at least half of the other pairs, namely hypervigilance and exaggerated startle response, and intrusions and difficulties falling asleep. Emotional and physical cue reactivity showed the highest centrality and trauma-related amnesia the lowest. Overall, this underlines the high relevance of re-experiencing symptoms in traumatized refugees with and without a PTSD diagnosis. The most important limitation of our study is the small sample size, resulting in a low power and limited robustness of our findings. The findings should therefore be interpreted with great care. Replication of our study with a larger sample size and further research with longitudinal data is crucially needed in future.

## Supplementary Material

Supplementary material

## References

[CIT0001] American Psychiatric Association (2013). *Diagnostic and statistical manual of mental disorders* (5th ed.). Washington, DC: Author.

[CIT0002] Armour C, Fried E. I, Deserno M. K, Tsai J, & Pietrzak R. H. (2017). A network analysis of DSM-5 posttraumatic stress disorder symptoms and correlates in US military veterans. *Journal of Anxiety Disorders*, 45, 49–10. 27936411 10.1016/j.janxdis.2016.11.008

[CIT0003] Bontempo R. (1993). Translation fidelity of psychological scales an item response theory analysis of an individualism-collectivism scale. *Journal of Cross-Cultural Psychology*, 24(2), 149–166. doi:10.1177/0022022193242002

[CIT0004] Borsboom D., & Cramer A. O. (2013). Network analysis: An integrative approach to the structure of psychopathology. *Annual Review of Clinical Psychology*, 9, 91–121. doi:10.1146/annurev-clinpsy-050212-185608 23537483

[CIT0005] Bryant R. A., Creamer M., O’donnell M., Forbes D., McFarlane A. C., Silove D., & Hadzi-Pavlovic D (2017). Acute and chronic posttraumatic stress symptoms in the emergence of posttraumatic stress disorder: A network analysis. *JAMA* *Psychiatry* 74(2), 135–142. 10.1001/jamapsychiatry.2016.3470 28002832

[CIT0006] Cramer A. O. J., Waldorp L. J., van der Maas H. L. J., & Borsboom D. (2010). Comorbidity: A network perspective. *Behavioral and Brain Sciences*, 33(2–3), 137–150. doi:10.1017/S0140525X09991567. 20584369

[CIT0007] De Fouchier C., Blanchet A., Hopkins W., Bui E., Ait-Aoudia M., & Jehel L. (2012). Validation of a french adaptation of the harvard trauma questionnaire among torture survivors from sub-saharan african countries. *European Journal of Psychotraumatology*, 3, 19225. doi:10.3402/ejpt.v3i0.19225 PMC3517723 23233870

[CIT0008] Ekstrøm M., Carlsson J., Sonne C., & Mortensen E. (2016). Stress management versus cognitive restructuring: A randomized clinical study on traumatized refugees. *European Psychiatry*, 33, S399–S400. doi:10.1016/j.eurpsy.2016.01.1437

[CIT0009] Epskamp S, Borsboom D, & Fried E. I (2017). Estimating psychological networks and their stability: a tutorial paper. doi: 10.3758/s13428-017-0862-1 PMC5809547 28342071

[CIT0010] Epskamp S., Cramer A. O., Waldorp L. J., Schmittmann V. D., & Borsboom D. (2012). qgraph: Network visualizations of relationships in psychometric data. *Journal of Statistical Software*, 48(4), 1–18. doi:10.18637/jss.v048.i04

[CIT0011] Foa E. B., Cashman L., Jaycox L., & Perry K. (1997). The validation of a self-report measure of posttraumatic stress disorder: The posttraumatic diagnostic scale. *Psychological Assessment*, 9(4), 445. doi:10.1037/1040-3590.9.4.445

[CIT0012] Fried E. I. (2015). Problematic assumptions have slowed down depression research: Why symptoms, not syndromes are the way forward. *Frontiers in Psychology*, 6, 309. doi:10.3389/fpsyg.2015.00309 25852621 PMC4369644

[CIT0013] Fruchterman T. M., & Reingold E. M. (1991). Graph drawing by force‐directed placement. *Software: Practice and Experience*, 21(11), 1129–1164.

[CIT0014] Hofmann S. G., Curtiss J., & McNally R. J. (2016). A complex network perspective on clinical science. *Perspectives on Psychological Science*, 11(5), 597–605. doi:10.1177/1745691616639283 27694457 PMC5119747

[CIT0015] IBM Corp (Released 2014). *SPSS statistics for macintosh, version 23.0*. Armonk, NY: IBM Corp.

[CIT0016] Knaevelsrud C., & Müller J. (2008). *Multilingual computer assisted self-interview (MultiCASI)*. Heidelberg: Springer.

[CIT0017] McNally R. J. (2016). Can network analysis transform psychopathology? *Behaviour Research and Therapy*, 86, 95–104. doi:10.1016/j.brat.2016.06.006 27424882

[CIT0018] McNally R. J., Robinaugh D. J., Wu G. W., Wang L., Deserno M. K., & Borsboom D. (2015). Mental disorders as causal systems a network approach to posttraumatic stress disorder. *Clinical Psychological Science*, 3(6), 836–849. doi:10.1177/2167702614553230

[CIT0019] Mollica R. F., Caspi-Yavin Y., Bollini P., Truong T., Tor S., & Lavelle J. (1992). The harvard trauma questionnaire: Validating a cross-cultural instrument for measuring torture, trauma, and posttraumatic stress disorder in indochinese refugees. *The Journal of Nervous and Mental Disease*, 180(2), 111–116. 1737972

[CIT0020] Mollica R. F., McInnes K., Pham T., Fawzi M. C. S., Murphy E., & Lin L. (1998). The dose-effect relationships between torture and psychiatric symptoms in vietnamese ex-political detainees and a comparison group. *The Journal of Nervous and Mental Disease*, 186(9), 543–553. 9741560 10.1097/00005053-199809000-00005

[CIT0021] Momartin S., Silove D., Manicavasagar V., & Steel Z. (2004). Comorbidity of PTSD and depression: Associations with trauma exposure, symptom severity and functional impairment in bosnian refugees resettled in australia. *Journal of Affective Disorders*, 80(2), 231–238. doi:10.1016/S0165-0327(03)00131-9 15207936

[CIT0022] Neuner F., Schauer M., Klaschik C., Karunakara U., & Elbert T. (2004). A comparison of narrative exposure therapy, supportive counseling, and psychoeducation for treating posttraumatic stress disorder in an african refugee settlement. *Journal of Consulting and Clinical Psychology*, 72(4), 579. doi:10.1037/0022-006X.72.4.579 15301642

[CIT0023] Nickerson A., Bryant R. A., Schnyder U., Schick M., Mueller J., & Morina N. (2015). Emotion dysregulation mediates the relationship between trauma exposure, post-migration living difficulties and psychological outcomes in traumatized refugees. *Journal of Affective Disorders*, 173, 185–192. doi:10.1016/j.jad.2014.10.043 25462415

[CIT0024] Opsahl T., Agneessens F., & Skvoretz J. (2010). Node centrality in weighted networks: Generalizing degree and shortest paths. *Social Networks*, 32(3), 245–251. doi:10.1016/j.socnet.2010.03.006

[CIT0025] Orth U., & Wieland E. (2006). Anger, hostility, and posttraumatic stress disorder in trauma-exposed adults: A meta-analysis. *Journal of Consulting and Clinical Psychology*, 74(4), 698. doi:10.1037/0022-006X.74.4.698 16881777

[CIT0026] Schick M., Zumwald A., Knöpfli B., Nickerson A., Bryant R. A., Schnyder U., … Morina N. (2016). Challenging future, challenging past: The relationship of social integration and psychological impairment in traumatized refugees. *European Journal of Psychotraumatology*, 7, 28057. doi:10.3402/ejpt.v7.28057 PMC4756625 26886484

[CIT0027] Schnyder U., Müller J., Morina N., Schick M., Bryant R. A., & Nickerson A. (2015). A comparison of DSM‐5 and DSM‐IV diagnostic criteria for posttraumatic stress disorder in traumatized refugees. *Journal of Traumatic Stress*, 28(4), 267–274. doi:10.1002/jts.22023 26194738

[CIT0028] Silove D., Liddell B., Rees S., Chey T., Nickerson A., Tam N., … Steel Z. (2014). Effects of recurrent violence on post-traumatic stress disorder and severe distress in conflict-affected timor leste: A 6-year longitudinal study. *The Lancet Global Health*, 2(5), e293–e300. doi:10.1016/S2214-109X(14)70196-2 25103168

[CIT0029] Steil R., & Ehlers A. (2000). Dysfunctional meaning of posttraumatic intrusions in chronic PTSD. *Behaviour Research and Therapy*, 38(6), 537–558. 10846804 10.1016/s0005-7967(99)00069-8

[CIT0030] Terluin B., De Boer M. R., & de Vet H. C. (2016). Differences in connection strength between mental symptoms might be explained by differences in variance: Reanalysis of network data did not confirm staging. *Plos One*, 11(11), e0155205. doi:10.1371/journal.pone.0155205 27880771 PMC5120783

[CIT0031] Tibshirani R. (1996). Regression shrinkage and selection via the lasso. *Journal of the Royal Statistical Society. Series B (Methodological)*, *58*(1), 267–288.

[CIT0032] Turner S. W., Bowie C., Dunn G., Shapo L., & Yule W. (2003). Mental health of kosovan albanian refugees in the UK. *The British Journal of Psychiatry*, 182(5), 444–448. 12724249

[CIT0033] UNHCR (2016). Global trends: Forced displacement in 2015. Retrieved 6 21, 2016. http://www.unhcr.org/statistics/unhcrstats/576408cd7/unhcr-global-trends-2015.html

[CIT0034] World Health Organization (1992). *The ICD-10 classification of mental and behavioural disorders: Clinical descriptions and diagnostic guidelines*. Geneva: World Health Organization.

