# Supplementary material for: Symptoms of posttraumatic stress disorder in a clinical sample of refugees: a network analysis
Source: Eur J Psychotraumatol. 2017 May 16;8(sup3):1318032. doi: 10.1080/20008198.2017.1318032 (PMC5639426; doi:10.1080/20008198.2017.1318032)
Supplement: Supplementary material [file ZEPT_A_1318032_SM8850.pdf]

The figures presented in this supplementary materials file are the resulting figures of the accuracy and stability analyses described in the main manuscript. The analyses were conducted using the R-package *bootnet, following* the recommendations of a tutorial paper on the use of bootnet (Epskamp et al., 2016).

Figure S1: Bootstrap edge weights difference test between non-zero estimated edge-weights in the network of 20 DSM-5 PTSD symptoms shown in Figure 1.

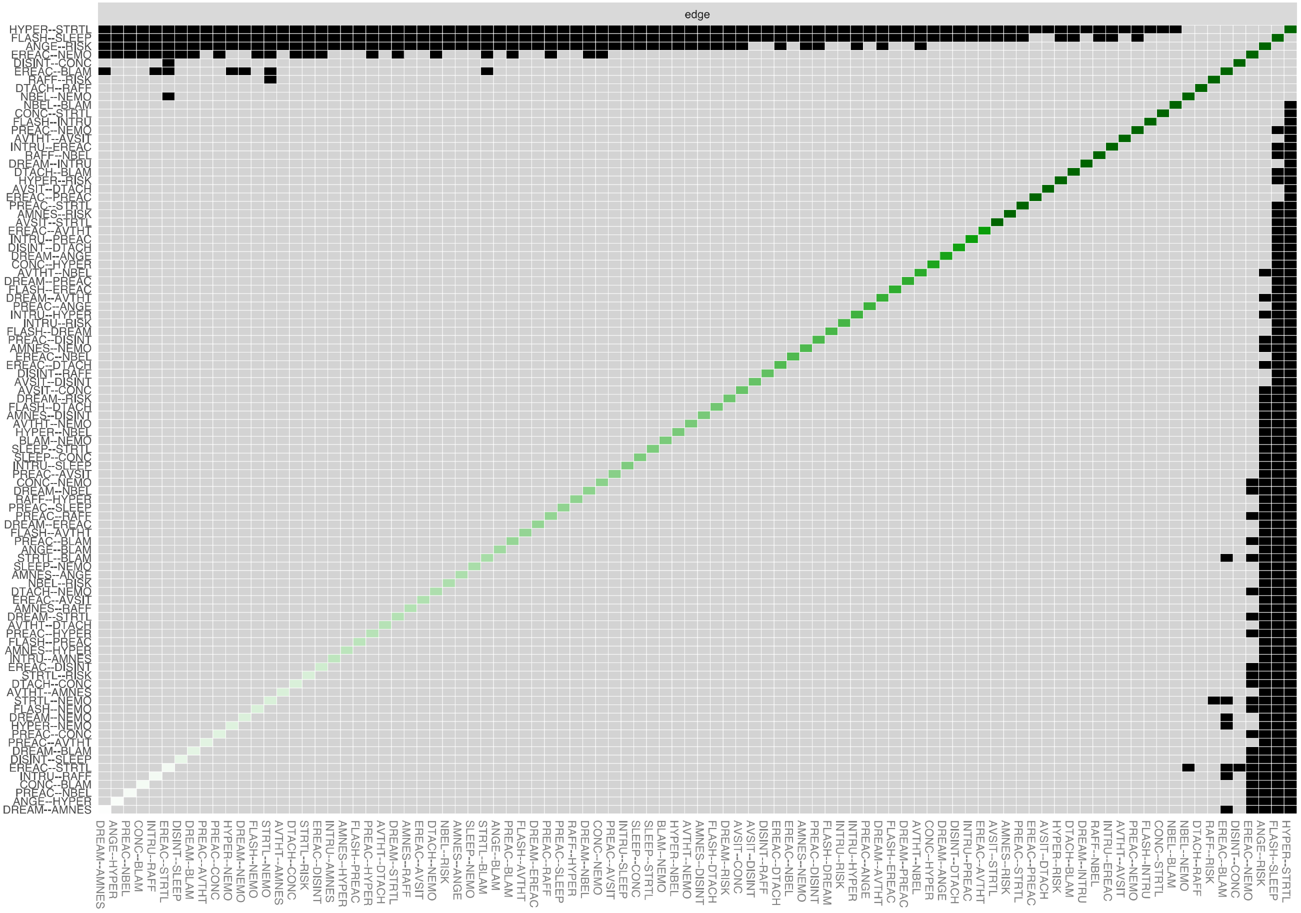

Notes:

Black boxes indicate a significant difference between two edges, gray indicate a non-significant difference. The color of the boxes (ranging from white to green) corresponds to the thickness of the edge in Figure 1. Correction for multiple testing was not available at the time of the analysis. PTSD: Posttraumatic stress disorder; DSM-5: Diagnostic and Statistical Manual of Mental Disorders, Fifth Edition

Figure S2: Bootstrap node strength difference test between node strength for the nodes of the network of 20 DSM-5 PTSD symptoms shown in Figure 1.

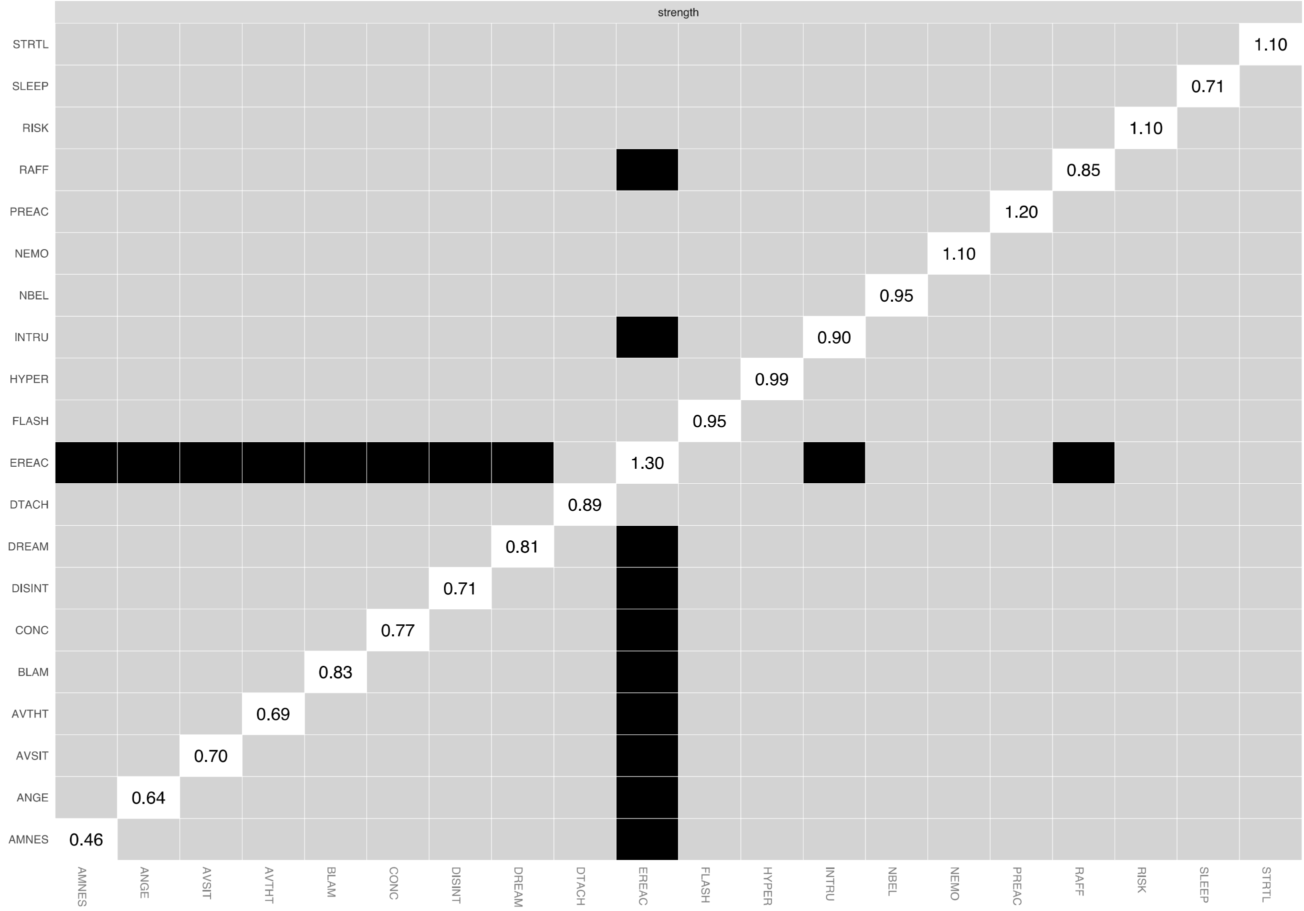

Notes:

Black boxes indicate a significant difference between two edges, gray indicate a non-significant difference. The number in the white boxes corresponds to the value of node strength. Correction for multiple testing was not available at the time of the analysis. PTSD: Posttraumatic stress disorder; DSM-5: Diagnostic and Statistical Manual of Mental Disorders, Fifth Edition

R code used

```
#######################################################################
#                                                                     #
#    Symptoms of posttraumatic stress disorder in a clinical sample of refugees    #
#                          - A Network Analysis                       #
#                                                                     #
#######################################################################



library("qgraph")
library("bootnet")



#### 1. Data
### 1.1 Read data from csv file
PTSD <- read.csv(„PTSD.csv“, sep=";", na.strings="99")



#### 2. PTSD Symptom Network
### 2.1 Estimation and visualization of network structure

## 2.1.1 By using qgraph
PTSD.cor <- cor_auto(PTSD)
EBICgraph <- EBICglasso(PTSD.cor, n = nrow(PTSD))
qgraph(EBICgraph, layout = "spring")


## 2.1.2 By using bootnet, Figure 1
Network <- estimateNetwork(PTSD, default = "EBICglasso")
plot(Network, layout = "spring", labels = TRUE, filename="Figure_1",
filetype='pdf')
```

## 2.2 Plotting Centrality, Figure 2

```
centralityPlot(EBICgraph)
```

#### 3. Robustness analysis

### 3.1 Edge weight bootstrap, Figure 3

```
bootEDGE <- bootnet(Network, nBoots = 1000)
plot(bootEDGE, labels = FALSE, order = "sample")
```

### 3.2 Node dropping bootstrap, Figure 4

```
bootNODE <- bootnet(Network, nBoots = 1000, type = "node")
plot(bootNODE)
```

### 3.3 Calculation of CorCofficient

```
corStability(bootNODE)
```

3.4 *Significance testing edge weights, Figure S1*

```
plot(bootEDGE, "edge", plot = "difference", onlyNonZero = TRUE, order = "sample")
```

3.5 *Significance testing node strength, Figure S2*

```
plot(bootEDGE, "strength")
```

References

Epskamp, S., Borsboom, D., & Fried, E. I. (2016). Estimating psychological networks and their stability: a tutorial paper. *Arxiv Preprint (ID 160408045) 1–34.*
